# Neural Correlates of the Appraisal of Attachment Scenes in Healthy Controls and Social Cognition—An fMRI Study

**DOI:** 10.3389/fnhum.2016.00345

**Published:** 2016-07-05

**Authors:** Karin Labek, Roberto Viviani, Elke R. Gizewski, Michael Verius, Anna Buchheim

**Affiliations:** ^1^Department of Clinical Psychology, Institute of Psychology, University of InnsbruckInnsbruck, Austria; ^2^Department of Psychiatry and Psychotherapy III, University of UlmUlm, Germany; ^3^Department of Neuroradiology, Medical University InnsbruckInnsbruck, Austria; ^4^Neuroimaging Research Core Facility, Innsbruck Medical UniversityInnsbruck, Austria

**Keywords:** attachment, fMRI, social cognition, mentalizing, temporo-parietal-junction

## Abstract

The human attachment system is activated in situations of danger such as potential separation, threats of loss of a significant other and potential insecurity on the availability of the attachment figure. To date, however, a precise characterization of the neural correlates of the attachment system in healthy individuals is lacking. This functional magnetic resonance imaging (fMRI) study aims at characterizing the distinctive neural substrates activated by the exposure to attachment vs. non-attachment scenes. Healthy participants (*N* = 25) were presented scenes from the Adult Attachment Projective Picture System (AAP), a validated set of standardized attachment-related pictures extended by a control picture stimulus set consisting of scenes without attachment-related content. When compared to the control neutral pictures, attachment scenes activated the inferior parietal lobes (IPLs), the middle temporal gyrus (MTG), and the anterior medial prefrontal cortex (mPFC). These areas are associated with reasoning about mental representations, semantic memory of social knowledge, and social cognition. This neural activation pattern confirms the distinctive quality of this stimulus set, and suggests its use as a potential neuroimaging probe to assess social cognition/mentalizing related to attachment in healthy and clinical populations.

## Introduction

The human attachment system is a behavioral system that is responsible for triggering specific responses when situations arise that signal potential physical or psychological danger or stress. In this respect, the human attachment system differs from other related innate attachment systems, such as the caregiving, affiliative, and romantic/sexual systems. Attachment theory is rooted within an evolutionary context and may be viewed as one of the motivational systems that have the function to maximize the chances of survival. When activated, the attachment system motivates behaviors that aim at responding the child’s need for protection or support, such as seeking the proximity of the caregiver (Bowlby, 1969, 1973, 1980).

Several neuroimaging studies have investigated the neural correlates of the caregiving (e.g., Bartels and Zeki, 2004; Leibenluft et al., 2004; Nitschke et al., 2004; Lenzi et al., 2009; Riem et al., 2012; Wittfoth-Schardt et al., 2012), the affiliative (e.g., Vrtička et al., 2008) and the romantic/sexual system (e.g., Bartels and Zeki, 2000, 2004; Gillath et al., 2005; Coan et al., 2006).

These studies have used various approaches to identify the neural correlates of distinctive attachment-related systems, such as pictures or video clips of one’s own vs. unknown children, pictures of romantic partners, have one’s hand held while being subjected to painful stimuli, or thinking about relationship scenarios. Notwithstanding the differences in methods and aims, several studies have reported a shared neural activity for the romantic and maternal attachment in regions associated with reward and motivation (ventral striatum, the putamen and the globus pallidus) and affective processing (orbitofrontal cortex).

However, much less is known about the neural correlates of the attachment system related to own representations. In the present study, we investigated the neural substrates activated by appraising situations that potentially activate the attachment system in the healthy adult.

In the child, the attachment system is activated on the one hand by attachment-related threats (for example, when infants are separated from their caregivers), and on the other hand by situations that are inherently threatening or increase the likelihood of danger (such as isolation, illness, injury; see Bowlby, 1969, 1973, 1980). In the adult, the attachment system may be activated by attachment-related stress, such as the loss of a loved one, and by a wider set of emotional or interpersonal situations. This activation is mediated by mental representations or schemata that encode past experiences of interactions with attachment figures in times of threat, of the availability or the intentions of attachment-figures, of the capacity of the attachment-figure to respond to our needs. Thus, according to attachment theory, in the adult responses to stimuli of social nature may also be organized by the attachment system when these stimuli are appraised through the appropriate schemata.

This fact is exploited by diagnostic instruments, such as the Adult Attachment Projective Picture System (AAP; George and West, 2001, 2012; Buchheim et al., 2006, 2008), that aim at characterizing in the adult the quality of the responses triggered by the activation of the attachment system. In the AAP interview, individuals are presented with scenes depicting attachment relationships or potential relationships in the presence of figural elements alluding to threatening conditions such as illness, separation, loneliness, or death. The quality of the attachment-related response of the individual is assessed through a semi-structured interview that measures the individual’s attachment representations activated by these scenes.

**Figure 1:**
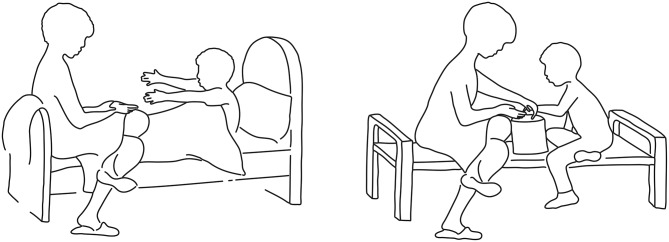
**Example of an original attachment-related scene of the Adult Attachment Projective Picture System (AAP) (left) and the newly created corresponding control scene (right)**.

To characterize the neural substrates activated by the exposure to the attachment scenes of the AAP and that are involved in processing these stimuli we developed a new set of control pictures that depicted almost the identical scenes, except for the replacement of the threatening figural elements with others of a more neutral nature (for example, see Figure 1). In developing these control pictures we made every effort to create scenes portraying social interactions and in which individuals appeared as possessing a purpose and/or mental activity, as in the original AAP scenes. The function of these control pictures was not one of offering impoverished scenes of human interaction, or individuals without a discernible mental content; instead, we attempted to recreate scenic elements of similar complexity as in the original AAP set, but without the reference to the cues that may activate the attachment system. Activated neural correlates were sought that emerged in the comparison between the exposure to the attachment scenes of the AAP diagnostic interview and the modified neutral scenes as a control condition.

The present study belongs to the set of studies that investigate the neural correlates of the own representations of attachment with respect to attachment related threats or loss of the reference person and insecurity about its permanence or availability (Buchheim et al., 2006, 2008, 2012, 2013). To date, these studies have instructed the participants to tell narratives to the AAP pictures in the functional magnetic resonance imaging (fMRI) environment or they used individualized text passages from the AAP interview to activate the attachment system. These studies intended to characterize differences between healthy participants and patient groups, such as depressives or individuals suffering from borderline personality disorder at a neural level. While these studies have successfully identified differences between healthy controls and patients, it has been difficult to interpret the data univocally in the absence of an adequate control stimulus set. Therefore, the present study is also meant as a methodological development to achieve improved specificity in the detection of the substrates associated with the attachment-related cues.

One issue raised by these studies is the involvement of areas, such as the right temporo-parietal-junction (TPJ; Buchheim et al., 2008), which in other studies has been shown to be part of a network underlying social cognition, and is often activated in response to social inputs, like social interactions (Frith and Frith, 2003). In the studies of the human attachment system (Buchheim et al., 2008), these areas are more often active in the presence of scenes depicting two interacting individuals (dyadic scenes) than in scenes showing only one person (monadic scenes). In attachment theory, social cognition abilities are considered essential to interact suitably with the attachment figure. These abilities are needed to make appropriate attributions about the mental states of others that may explain their behavior in the interaction. Especially in times of need or in the presence of threats, what individuals know about the mental state of attachment figures is important to behave so as to obtain proximity and care (see Bowlby, 1969, 1973, 1980). Recruitment of brain areas associated with social cognition (e.g., temporal poles, TPJ, posterior superior temporal sulcus), has been reported in few other studies of attachment (Leibenluft et al., 2004). The more complex interpersonal nature/information of the dyadic scenes may indicate the need for greater cognitive processing and could be particularly effective in eliciting schematic representations of social interactions.

A further issue arising from the comparison with studies of different forms of attachment is the predominantly negative valence of stimuli activating the human attachment system. This contrasts with the generally appetitive nature of stimuli activating the affiliative, caregiving, and romantic systems. Processing of stimuli of negative emotional valence is often associated with the activation of the amygdala (Davis and Whalen, 2001; Adolphs et al., 2005). This activation, however, may be related to the generally high arousing nature of these stimuli (Adolphs, 2010). Strathearn and Kim (2013) reported amygdala responses to positively or negatively valenced cues (own vs. unknown child, happy, sad, neutral faces) modulated by personal relevance. To address this issue, we characterized the amount of negative valence and arousal in our stimulus set relative to other sets commonly used in neuroimaging studies, and examined the activation of the amygdala with region-of-interest (ROI) analyses.

## Materials and Methods

The study protocol was approved by the Ethical Committee of the Medical University of Innsbruck and was in compliance with National legislation, the principles expressed in the Declaration of Helsinki, and the Code of Ethical Principles for Medical Research Involving Human Subjects of the World Medical Association. All participants gave written informed consent.

### Participants

A sample of *N* = 25 healthy volunteers were recruited at Leopold-Franzens University in Innsbruck (age mean/SD 22.7/1.8, age range: 19–26, 12 females, see Table 1). Depressiveness was measured with a computerized German Version of the Centre for Epidemiologic Studies Depression Scale (CES-D, Radloff, 1977; German Version: Hautzinger and Bailer, 1993). The present and long-lasting anxiety levels were assessed by the State-Trait Anxiety Inventory (STAI, Spielberger et al., 1970; German Version: Laux et al., 1981) All participants had scores in a subclinical range. Finally participants were assessed with the International Neuropsychiatric Interview (M.I.N.I. 5.0.0, Sheehan et al., 1998, German version) to exclude previous psychiatric or psychological illnesses.

**Table 1:** **Participants**.

	*n = 25*
Age mean (std. dev.)	22.7 (1.8)
Female (%)	12 (48)
Male (%)	13 (52)
ADS (std. dev.)	10.08 (10.39)
STAI-S (std. dev.)	35.60 (11.55)
STAI-T (std. dev.)	35.68 (11.36)

### Stimulus Material

The stimulus material consisted of attachment-related pictures (AAP^©^ George and West, 2012) and their modified neutral counterparts as a control condition. The AAP, an established and validated interview to assess attachment representations, is comprised of eight black and white line drawn pictures: seven attachment-related drawings and one neutral scene used as a warm-up. The attachment-related pictures are designed to activate the attachment system and to elicit mental engagement with attachment-related experiences (such as loneliness, helplessness, loss following illness, separation, solitude, death, and abuse). These drawings contain no facial expressions, and figures are drawn in summary strokes without precise details, but include the essential characteristics to recognize the attachment context. Drawings portray adult-adult dyads, adult-child dyads, adults alone, and children alone and capture attachment across the life span, infancy to old age.

For the control condition, pictures of attachment scenes from the AAP (George and West, 2012) were expanded with a set of carefully matched control scenes of non-attachment “neutral” character (Figure 1). The control battery consisted of eight line drawings of non-attachment pictures developed by the first author. In the control set, allusions to loneliness, potential rejection, death or illness, etc., were removed while replicating objects/characters, spatial structure and object configuration as closely as possible. Therefore, elements such as the monadic or dyadic nature of the scene, the age of the individuals depicted in it, etc., was precisely matched in the control picture set. The control scenes had the same size and were aligned with the corresponding AAP pictures. Care was given to prepare all pictures, attachment-related and non-attachment related, with the same stroke width, thickness, and size to make them formally identical. Since the original AAP warm-up scene contains no attachment-related content, we created a matched attachment-related scene (the picture of two children playing ball was transformed into a scene of potential separation). In total, there were 16 pictures, eight for each condition.

### Validation of the Stimulus Material

To validate the stimulus set, a preliminary behavioral study was conducted on a separate group of 97 healthy subjects (age mean/SD 23.15/5.12, 72 females). In this preliminary study, all scenes (attachment-related and control) were rated according to the dimensions valence and arousal through the self-assessment manikin (SAM) scales developed by Bradley and Lang (1994). This pictorial rating system records self-assessments of experienced emotion, rated according to icons depicting a 5-point scale along the dimensions affective valence and arousal. This rating system was developed to assess sets of standardized stimuli for experimental investigations that are widely used in neuroimaging investigations of emotion (International Affective Picture System [IAPS], Lang et al., 2008). Hence, the collection of these validation data makes it possible to compare the present stimulus set with other visual stimulus sets used in the neuroimaging literature.

**Figure 2:**
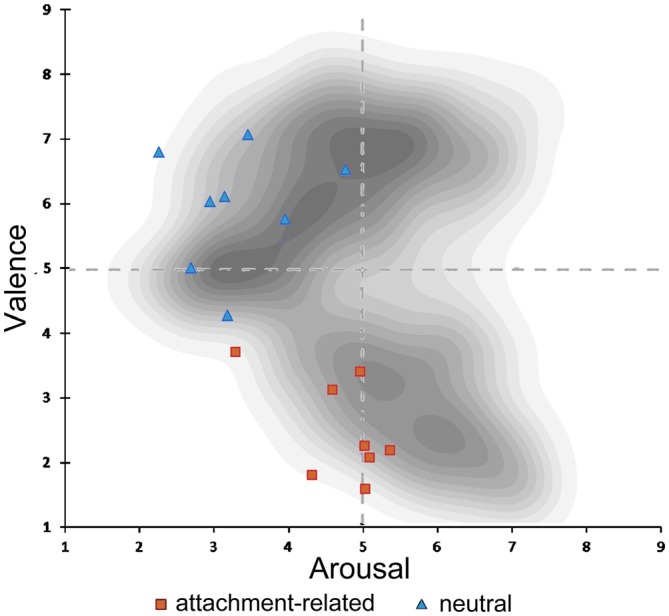
**Illustration of the two-dimensional affective space of the International Affective Picture System (IAPS) pictures.** In shades of gray, density of pictures in the original IAPS database in relation to valence and arousal. Rating of valence and arousal of the attachment-related scenes (red dots); rating of the neutral scenes (blue dots).

Valence ratings of the attachment-related pictures (mean score = 1.85, SD = 1.00) were significantly more negative in comparison to the ratings of neutral pictures (mean score = 3.75, SD = 0.98; *T* = −37.971, *p* < 0.000). Attachment-related pictures were rated of higher arousal (mean score = 3.06, SD = 1.18) than neutral pictures (mean score = 2.28, SD = 1.08; *T* = 13.577, *p* < 0.000; Knoll, 2015). These results confirmed that the new control picture-set significantly differed from the original AAP-set. However, it also suggested that the attachment-related pictures differed from typical negatively valenced visual stimuli used in previous neuroimaging studies. In those studies, the arousal of negatively valenced scenes may be higher than the arousal of the attachment-related scenes in the AAP set. Figure 2 shows the combination of the arousal and valence scores of the pictures in our set, plotted in a two-dimensional affective space representing the valence-arousal relationship. The gray dots show the arousal and valence of the IAPS, which is the source of material often used in neuroimaging studies (Lang et al., 2008). This plot shows the association of extreme valence scores (either positive or negative) with high arousal scores in the IAPS. In our picture set, negative emotional scores (in red) were not associated with arousal values substantially above the middle line. As in the IAPS set, there was an association of degree of negative valence in the attachment-related pictures with the level of arousal; however, the arousal levels were consistently less than what one would expect for pictures of comparable negative valence.

### fMRI Data Acquisition, Preprocessing, and Statistical Modelling

Participants were placed supine in the scanner, wearing earplugs to muffle noise. Head fixation was assured through a foam rubber device mounted on the head coil. Data were acquired using a T2*-weighted echo planar imaging (EPI) sequence on a 3T Siemens Verio scanner (repetition time (TR)/echo time (TE) 2900/30 ms, transversal acquisitions, phase encoding anterior-posterior, field of view (FOV) 1260 * 1260, slice thickness 2.5 mm with a gap of 1 mm, giving a voxel size of 1.88 × 1.88 × 3.5 mm, flip angle 90°, pixel bandwidth 1680 Hz). Data were acquired in two separate runs, each of which contained eight trials in which a scene was shown for 15 s, followed by a fixation cross for further 15 s Participants were instructed to pay attention to the persons depicted in scenes. Each run contained eight trials, equally divided into two of the two types of scenes (attachment-related or control). Scenes constituting the control version of the original attachment-related AAP scenes were shown in different runs, so that each run contained exposure to the whole set of eight scenes (attachment-related or control). To avoid systematic effects, the sequence in which scenes were recruited in the two runs was systematically changed across subjects.

Data were analyzed after realignment, normalization (resampling size, 2 mm isotropic) and smoothing (8 mm full width at half maximum (FWHM)) with the software package SPM8^1^. Contrasts of interest were estimated at the first level within each participant separately, and brought to the second level to account for the random effect of subjects. We report effects at *p* < 0.05, cluster-level corrected, with clusters defined by the threshold *p* < 0.001, uncorrected.

The data were analyzed with a model in which the blocks during which pictures were displayed were convolved with a standard blood oxygen level dependent (BOLD) hemodynamic function to account for the delay of onset of the signal. A design matrix was constructed containing two factors of two levels each: attachment/control and monadic/dyadic. Appropriate contrasts were taken to the second level to estimate the effects of attachment and its interaction with the monadic or dyadic nature of the pictures.

## Results

**Figure 3:**
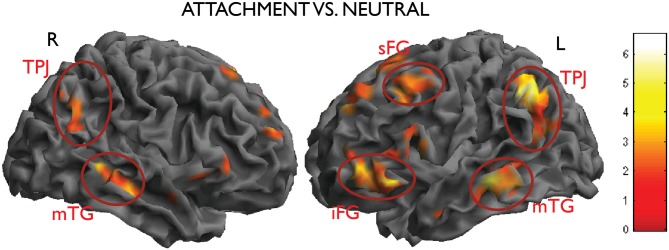
**Activations from the main contrast of interest attachment vs. neutral (left inferior parietal lobe (IPL)/temporo-parietal junction (TPJ), middle temporal gyrus (MTG), the left superior and inferior frontal gyri (SFG and IFG), picture on the right side; right IPL and MTG, picture on the left side).** Data thresholded at *p* < 0.005, uncorrected, for display purposes.

In the contrast attachment vs. neutral we found a statistical significant activation in the left inferior parietal lobe (IPL)/temporo-parietal junction (TPJ) (MNI coordinates *x, y, z* = −46, −52, 42, *t* = 6.67, number of voxels in cluster *k* = 833, *p* < 0.001, cluster-level corrected), in the left middle temporal gyrus (MTG) (*x, y, z* = −66, −24, −12, *t* = 5.10, *k* = 258, *p* = 0.005). These clusters were accompanied by less pronounced activations on the opposite side, which failed to reach significance after correction (IPL: *x, y, z* = 62, −54, 36, *t* = 4.69, *k* = 54, n.s.; MTG, *x, y, z* = 52, −20, −16, *t* = 4.98, *k* = 64, n.s.). In the prefrontal lobes, the left superior and inferior frontal gyri (SFG and IFG) and the anterior medial prefrontal cortex (mPFC) were also activated in this contrast (*x, y, z* = −42, 16, 56, *t* = 5.40, *k* = 273, *p* = 0.004; *x, y, z*; Figure 3).

**Figure 4:**
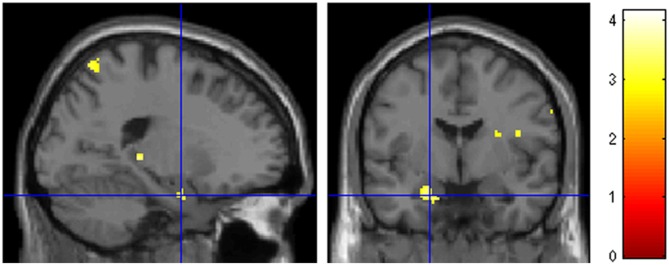
**Deactivation of the amygdala in the main contrast attachment vs. neutral pictures.** Same display threshold as in the previous Figure (*p* < 0.005).

A question of interest was the involvement of the amygdala, given the combination of marked negative emotional valence and low arousal values of these pictures demonstrated by the behavioral validation study (see Figure 2 in the “Materials and Methods” Section). There was a bilateral light deactivation of the amygdala in the contrast attachment vs. neutral, which however was not significant after correction (*x, y, z* = −24, −2, −24, *t* = −3.55; *x, y, z* = 22, 0, −22, *t* = −2.54; Figure 4).

A second contrast of interest investigated the modulation of the effect of attachment by type of scenes (dyadic or monadic). In the contrast attach vs. control within the dyadic scenes group, the right temporo-parietal region was activated more strongly (effect within dyadic scenes: *x, y, z* = 56, −52, 34, *t* = 5.75, *k* = 517, *p* < 0.001; effect within monadic scenes: *x, y, z* = 54, −64, 40, *t* = 3.84, *k* = 19, n.s.; interaction: *x, y, z* = 62, −32, 16, *t* = 4.33, *k* = 47, n.s; see Figure 3, top row). An interaction between the two factors attachment/neutral × dyadic/monadic was observed in the occipital lobes bilaterally (*x, y, z* = 48, −78, 6, *t* = 5.44, *k* = 123, *p* = 0.079; *x, y, z* = −40, −80, −6, *t* = 4.63, *k* = 139, *p* = 0.050).

**Figure 5:**
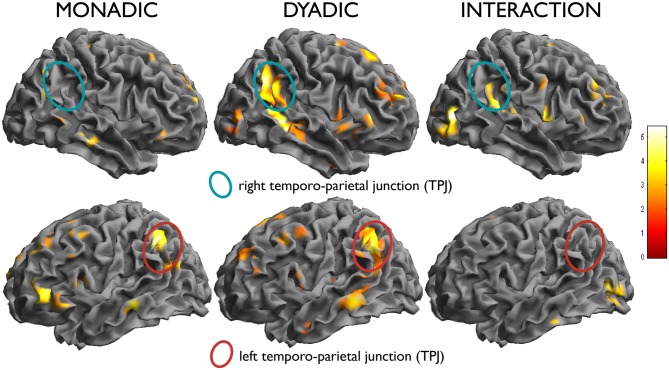
**In the left column, effect of the simple effect attach vs. control within monadic scenes, and in the middle column, within dyadic scenes.** Note the activation of the left temporo-parietal region in both (bottom row), and the selective activation of the right counterpart in the dyadic scenes (top row). In the right column, test of the interaction attachment/control × dyadic/monadic, showing the differential effect in the right TPJ.

There was no significant interaction in the opposite direction, i.e., for effects of attachment that were larger in the monadic than in the dyadic group. As one may see from Figure 5 (bottom row), this was due to effects of similar intensity within both picture types in the areas activated by attachment, such as the TPJ on the left. Also the apparent higher activation in the left IFG in the monadic group, visible in the bottom row of Figure 3, failed to achieve significance (*x, y, z* = −36, 38, 2, *t* = 3.19).

## Discussion

The main contrast of interest of our study revealed the activation of areas of predominantly associative nature, such as the IPL and the MTG, in association with attachment-related stimuli. These findings replicate those of previous studies in which the AAP pictures were used in comparisons between healthy participants and patients (Buchheim et al., 2006, 2008). Activations in the anterior insula have a similar interpretation but have been more explicitly associated with emotional representations (Gu et al., 2013). This pattern of activation contrasts with those commonly obtained in studies of negatively valenced visual stimuli, which are characteristically more arousing and activate limbic structures such as the amygdala (for a quantitative review, see Costafreda et al., 2008). The activation of these limbic structures has been shown to be related to the early automatic processing of sensory aspects of the stimulus (Morris et al., 1999), which trigger arousal levels favoring its attentional prioritization for further processing (Vuilleumier, 2005). The lack of recruitment of these circuits in the present study suggests that appraisal of attachment content recruited processes that differed from those typically associated with the exposure to negatively valenced stimuli in many previous neuroimaging studies. Activation of the amygdala in studies of attachment styles that used facial expression stimuli may have detected differences in arousal and emotional reactivity (Vrtička et al., 2008).

The most prominent among the association areas activated by the attachment-related content was the TPJ, a region that numerous previous studies have identified as a neural correlate of theory of mind tasks and social cognition (for reviews, see Gallagher and Frith, 2003; Saxe and Kanwisher, 2003; Decety and Lamm, 2007; Van Overwalle, 2009; Saxe, 2010; Carter and Huettel, 2013). Theory of mind is a construct that refers to the cognitive capacity to form a representation of other people’s beliefs and intentions (Premack and Woodruff, 1978; Baron-Cohen, 1997). Neuroimaging studies have investigated this construct through story comprehension tasks that require the correct attribution of beliefs after changes in the environment (Mar, 2011). Social cognition is a label that more generically refers to the adequate perception and evaluation of social stimuli and the processing of information about social interactions, including their affective components. Functional neuroimaging studies have associated social cognition processes with an extensive network of areas, which, beside the TPJ, also includes the middle temporal sulcus, the temporal poles, and the mPFC (Frith and Frith, 2012).

Neuroimaging studies of theory of mind have reported that left TPJ activations are more common when processing different subjective perspectives, whereas the right parietal junction was more specifically activated by processing states like beliefs (Perner et al., 2006). In the present study, dyadic pictures activated the TPJ on both sides, while monadic pictures activated this structure only on the left. This result is consistent with a more complex representation of beliefs and mental states in the presence of interactions between participants in the scenes, relative to pictures where the mental state of the individual in the scene could be inferred directly from pictorial elements such as her body posture (Peelen and Downing, 2007).

In attachment theory, the concept of mentalization refers to the capacity of the individual to understand one’s own and other people’s actions in the light of intentions, personal desires, needs or beliefs (Fonagy and Target, 2005). To predict behavior on the basis of mental states, the current literature divide mentalization processes in emotional and cognitive aspects. Emotional mentalizing abilities are referred to the ability to empathize emotionally like representations of intentions and believes. Emotional contagion/mentalization processes are thought to involve a network of brain area e.g., IFG and the IFL (Shamay-Tsoory, 2011) while cognitive mentalization processes activate brain regions like e.g., the TPJ (Saxe, 2006; Vrtička and Vuilleumier, 2012) or superior temporal sulcus (Frith and Frith, 2003). In the AAP interview, participants are invited to develop narratives over the motives, feelings and thoughts of the individuals depicted in the scenes. Activation of the neural substrates of social cognition in the present study suggests that the healthy participants in our sample spontaneously reacted to attachment scenes by elaborating such motives, especially in the presence of dyadic scenes.

Mentalization is an important individual trait that maybe associated with better affect- and self-regulation capacity (Fonagy and Luyten, 2009; Viviani et al., 2011). Dysfunction of self-regulation is crucial especially in the context of social relationships (Posner et al., 2002) Mentalization capacities in the adult are considered to develop from the internalization in childhood of the capacity for containment of the attachment figure. This containment enables the child to experience its own emotions without being overwhelmed by them (Fonagy et al., 1995; Bateman and Fonagy, 2004). In the theory underlying the AAI interview, this capacity is referred to as “reflective functioning” (Fonagy et al., 1998). Neuroimaging studies attempting to locate the neural correlates of mentalization have reported activations in the left MTG and IFG, two foci that were active also in the present study (Nolte et al., 2012).

In the alternative DSM-5 model for personality disorders (see “Results” Section), personality functioning (Criterion A) was included to assess the level of functional impairment associated with personality disorders (DSM-5; American Psychiatric Association, 2013). The level of personality functioning scale (LPFS; Bender et al., 2011) conceptualizes intrapersonal (identity, self-direction) and interpersonal (empathy, intimacy) components of the underlying personality structure. Hence, the ability to understand oneself and the social and internal world of others in terms of mental states are viewed as an essential trait of personality disorders. Here, working models of attachment theory and implicit representations of others represent a key theoretical and empirical issue (Skodol et al., 2011). Reflecting the increasing recognition of the importance of personality functioning, fMRI studies are beginning to apply assays eliciting activation in areas associated with representations of self (Doering et al., 2012) and empathy (Dziobek et al., 2008; Fan et al., 2011; Decety et al., 2013; Mier et al., 2013) to assess functional differences in personality disorders.

In summary, the present study provided evidence that exposure to the attachment scenes activated a cortical network that is recruited to represent the mental states of oneself and of others. These results are consistent with attachment theory, according to which the capacity to correctly interpret and process social cues from the attachment figure is important for responding appropriately in attachment relationships, thus ensuring security and proximity to the attachment figure. Our results suggest that while exposure to attachment-related themes, especially depicting interactions, specific processing takes place in brain areas associated with social cognition. The recruitment of social cognition-related areas while assessing attachment related pictures may be used in future studies to further explore its potential in detecting individual differences in mentalization capacities. By comparing the results of this and other functional assays, fMRI may contribute to clarifying the constructs involved in personality functioning.

## Author Contributions

The study was conceptualized by KL, AB, RV. The study setup and data collection were organized and conducted by KL, RV, ERG, MV. fMRI analyses was performed by KL and RV. KL and RV performed the statistical data analysis and contributed substantially to the result interpretation. AB and ERG provided important intellectual contribution in commenting and revising the manuscript. KL, RV, and AB wrote the manuscript and edited its final version.

## Conflict of Interest Statement

The authors declare that the research was conducted in the absence of any commercial or financial relationships that could be construed as a potential conflict of interest.

## Footnotes

^1^http://www.fil.ion.ucl.ac.uk/spm/
